# A Brief Training Program to Support the Use of a Digital Pill System for Medication Adherence: Pilot Descriptive Study

**DOI:** 10.2196/26213

**Published:** 2021-04-23

**Authors:** Peter R Chai, Maria J Bustamante, Georgia Goodman, Yassir Mohamed, Jesse Najarro, Matthew C Sullivan, Jose Castillo-Mancilla, Ryan P Coyle, Kenneth H Mayer, Rochelle K Rosen, Susan L Baumgartner, Pamela E Alpert, Edward W Boyer, Conall O'Cleirigh

**Affiliations:** 1 Department of Emergency Medicine Brigham and Women's Hospital Boston, MA United States; 2 The Koch Institute for Integrated Cancer Research Massachusetts Institute of Technology Cambridge, MA United States; 3 The Fenway Institute Boston, MA United States; 4 Department of Psychosocial Oncology and Palliative Care Dana Farber Cancer Institute Boston, MA United States; 5 Department of Psychiatry Massachusetts General Hospital Boston, MA United States; 6 Division of Infectious Disease Department of Medicine University of Colorado Anschutz Medical Campus Aurora, CO United States; 7 Department of Medicine Beth Israel Deaconess Medical Center Boston, MA United States; 8 Center for Behavioral and Preventive Medicine The Miriam Hospital Providence, RI United States; 9 Department of Behavioral and Social Sciences Brown University School of Public Health Providence, RI United States; 10 eTectRx Inc Gainesville, FL United States

**Keywords:** digital pill system, technology training, HIV prevention, PrEP, ingestible sensors, mobile phone

## Abstract

**Background:**

Digital pill systems (DPSs), which comprise ingestible radiofrequency sensors integrated into a gelatin capsule that overencapsulates a medication, can directly measure ingestion events.

**Objective:**

Teaching users to operate a DPS is vital to ensure the collection of actionable ingestion and adherence data. In this study, we aim to develop and pilot a training program, grounded in the Technology Acceptance Model, to instruct individuals on DPS operation.

**Methods:**

A two-part training program, comprising in-person and text message–based components, was used with HIV-negative men who have sex with men with nonalcohol substance use, who had enrolled in a 90-day pilot demonstration study using the DPS to measure adherence to pre-exposure prophylaxis. We assessed the number of responses to text check-ins, the number and types of episodes where technical support was requested, the resolutions of such issues, and engagement with the program over the study period. Participant feedback on the program was evaluated through qualitative user experience interviews.

**Results:**

A total of 15 participants were enrolled in and completed the program. Seven technical challenges related to DPS operations were reported across 5 participants. Most commonly, participants requested support connecting the wearable Reader device with their smartphone, charging the Reader, and operating the mobile app. A total of 6 issues were resolved asynchronously or in real time via phone; 1 required in-person evaluation and resolution. Preliminary qualitative findings indicate that both the in-person and remote follow-up components of the training program were perceived as acceptable. Suggested improvements included repeated DPS refresher sessions at in-person follow-up visits and enhanced written materials for the independent resolution of technological issues.

**Conclusions:**

A brief two-part DPS training program, drawing from individuals’ experiences and from the Technology Acceptance Model, can provide valuable insights for users. The program also identifies and addresses several areas of actual or potential challenges related to operating a DPS and allows for the resolution of such issues within the first week of DPS use.

## Introduction

Digital pill systems (DPSs) use ingestible electronic sensors incorporated into gelatin capsules that overencapsulate medications. Ingestible sensors are activated upon entry into the stomach and exposure to gastrointestinal fluid, transmitting key data about medication ingestion patterns in real time. These data can be viewed by clinicians and patients via web-based platforms and smartphone apps. The provision of such feedback linked to medication ingestion patterns represents a novel, cybernetic closed-loop system that allows users to consider their medication adherence in light of digital pill–recorded data [1,2]. Behavioral interventions that are respondent to real-time adherence data can then be delivered both synchronously or asynchronously as contingent reinforcement or corrective feedback, and the effects of these interventions can be objectively measured.

For men who have sex with men (MSM) with concomitant substance use, the use of once-daily tenofovir disoproxil fumarate/emtricitabine as pre-exposure prophylaxis (PrEP) for HIV prevention has been demonstrated to be highly efficacious; however, PrEP efficacy remains closely linked to adherence [3]. Post hoc analyses from clinical trials and demonstration projects around PrEP rollout have consistently demonstrated that MSM with substance use disorder experience the highest rates of nonadherence [4,5]. Techniques involving mobile apps, social media, and technological devices have been deployed among MSM to discover episodes of nonadherence and deliver interventions to help boost adherence behavior [6,7]. The use of such innovative technologies may help overcome barriers to engagement in PrEP care experienced by MSM and individuals with substance use disorder [8]. Among substance users, smart pill bottles, biosensors, mobile apps, and interactive voice response systems have been successfully deployed and demonstrated to be acceptable in the context of medication adherence measurement [9]. The development of a DPS provides the additional benefit of obtaining PrEP adherence data in real time, thereby creating an opportunity to present adherence support and tools to PrEP users at the moment when nonadherence occurs.

Ensuring consistent use of the DPS is vital to obtain actionable medication ingestion data that would enable real-time adherence interventions. Although several clinical trials have demonstrated the feasibility and acceptability of deploying a DPS to measure medication adherence, barriers to the adoption of this technology may exist [10-14]. The Technology Acceptance Model (TAM) [15-17] can serve as a theoretical framework for understanding and evaluating potential facilitators and barriers to the adoption and use of novel technology systems such as the DPS. In the TAM, users of a technology are asked to consider how they might use the system (perceived use) in the real world. Next, the overarching goals of the system and its intersections with perceived use are considered (intended use). Finally, users are asked how they would adopt the system in real-world situations (actual use) [15].

Our team conducted qualitative exploration of these 3 TAM constructs with HIV-negative MSM who use substances, which found that such individuals are accepting the idea of using a DPS to measure adherence to once-daily PrEP and are willing to engage with the technology [18]. Participants perceived the DPS to be a useful, innovative tool for measuring adherence and providing increased accountability and reassurance around their PrEP-taking patterns and behaviors. In addition, participants described a number of potential barriers to the process of learning to operate a DPS and barriers to their understanding of how to address potential technical challenges associated with the use of the technology [18]. Importantly, participants in these qualitative interviews reported that the wearable Reader device would be the largest barrier to operating the DPS, as users may forget to wear or charge the Reader before their ingestion, which could influence the accuracy of their adherence data. A brief in-person training program, combined with a period of remote follow-up, was reported to be an acceptable method for learning the skills required to operate the DPS [18]. Participants emphasized the importance of supervised hands-on training with the DPS, including testing the functionality of all components of the system before initiation, and a monitored follow-up period to assess the ongoing DPS operation.

Accordingly, we developed a brief training program with features addressing the 3 pillars of the TAM to teach individuals how to operate and incorporate the DPS into their daily routine. Our goal is to design a program that could first be taught to individuals who were naïve to the DPS and subsequently be delivered to DPS users by these individuals once they obtained a basic understanding of the DPS, to maximize its scalability and use for individuals using a DPS. We piloted this training program as part of a clinical trial that deployed a DPS to measure PrEP adherence over a 90-day period in HIV-negative MSM with nonalcohol substance use. Finally, we aim to solicit qualitative feedback via user experience interviews to understand the experiences of individuals who participated in the DPS training program.

## Methods

### Parent Study

The parent study consisted of a 90-day, open-label demonstration trial to evaluate the feasibility and acceptability of the DPS (ID Cap System, etectRx Inc) to measure PrEP adherence, in which digital pills for PrEP were deployed among HIV-negative MSM (N=15) older than 18 years who self-reported nonalcohol substance use (NCT03842436). Participants ingested digital pills once daily for 90 days and attended 5 study visits over a period of 3-4 months. In addition to DPS technology training, study visits consisted of laboratory work (to confirm eligibility for PrEP), a brief quantitative assessment, pill counts, reviews of DPS adherence data, blood draws to evaluate PrEP adherence, and provision of three 30-day supplies of digital pills. As part of this parent study, we developed a training program grounded in our initial qualitative work to ensure participants’ successful operation of the DPS over the course of the study, which is the focus of this study, and all adherence metrics from participants’ use of the DPS during the parent study will be reported elsewhere.

### Participants

A total of 15 participants enrolled in the parent study, a sample size that corresponded to the preliminary pilot nature of the study and our aim to demonstrate the feasibility and acceptability of the technology. All 15 participants were enrolled in the DPS training program. Participants met the following inclusion criteria: 18 years or older, cisgender MSM, self-reported use of nonalcohol substances in the past 6 months, currently taking PrEP, and qualifying laboratory tests for the use of PrEP (negative rapid HIV test, creatinine clearance ≥60 mL/min, evidence of hepatitis B immunization, and screening for sexually transmitted infections). Individuals were excluded if they did not speak English; self-reported living with HIV; were identified as transgender; had an estimated creatinine clearance <60 mL/min; were receiving active hepatitis B treatment; were taking proton pump inhibitors; had a history of Crohn disease or ulcerative colitis; had a history of bowel surgery, gastric bypass, or bowel stricture; had a history of gastrointestinal malignancy or radiation to the abdomen; did not own a smartphone; or were unable or unwilling to ingest the digital pill. This study was approved by the Fenway Health Institutional Review Board. A parent study was conducted between March 2019 and March 2020.

### Procedures

#### Training Program

**Figure 1:**
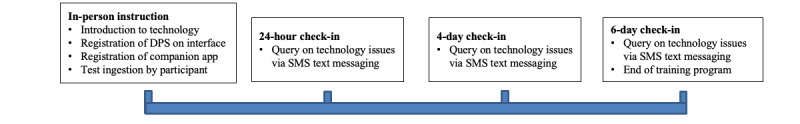
Overall structure of the digital pill system training program in-person session and remote follow-up. DPS: digital pill system.

The DPS training program consisted of 2 components: an in-person training session during the study enrollment visit and a remote follow-up period of text message–based exchanges for 6 days after enrollment (Figure 1). The genesis of this training program was grounded in formative qualitative data collected from MSM who evaluated the DPS and provided insights into the perceived use and intended use of the device [18]. The study team members first received training and information on the DPS and operation of the DPS directly from the manufacturer (etectRx Inc). Next, the study team members demonstrated competence with activating and operating the DPS, completing the web-based DPS registration process with sample participants and troubleshooting common potential technological errors. After demonstrating competence in the DPS operation, the principal investigator approved the study team members for conducting DPS training directly with the study participants.

**Figure 2:**
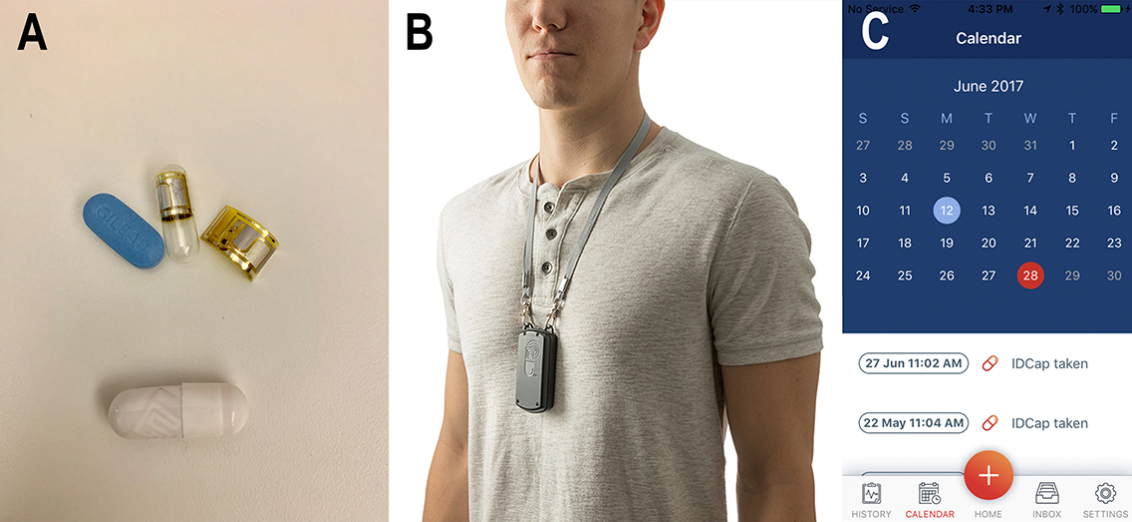
Components of the digital pill system. The digital pill system comprises an ingestible electronic sensor integrated into a gelatin capsule overencapsulating tenofovir disoproxil fumarate/emtricitabine (Truvada) as a digital pill (A). Upon ingestion, the digital pill is activated and emits a radiofrequency signal that is transmitted to a wearable Reader device (B). Ingestion data then flow from the Reader device to a smartphone app (C).

Before the in-person session, the study team registered each participant on the web-based DPS interface, electronically assigned a Reader device to them, and prepared a prescription for their initial 30-day supply of digital pills. Each digital pill consisted of a capsule dosage form containing an ingestible sensor and an emtricitabine 200 mg/tenofovir disoproxil fumarate 300 mg tablet for PrEP, with instructions to take one capsule once daily (Figure 2). During the in-person session, participants were introduced to each part of the DPS, including a transparent version of the digital pill, which was used to illustrate its internal components, the wearable Reader device and the companion mobile app. Participants were instructed on proper charging procedures for the Reader, the importance of maintaining a full charge on the Reader’s battery, and the process by which data would flow from the digital pill to the Reader and then to the cloud-based server.

Next, participants were instructed to download the smartphone app from either the Apple App Store or Google Play Store, depending on their device specifications. Following the installation of the mobile app, participants were instructed to activate the app by following in-app prompts and then pair the assigned Reader with the participant’s smartphone using a standard Bluetooth Low-Energy protocol (similar to how a smart speaker or smart watch pairs with a smartphone). Once the pairing process was complete, participants were asked to confirm that all DPS components were ready for use; they were then instructed to wear the Reader around their neck on a lanyard, open the mobile app on their smartphone, and ingest their first digital pill. A successful DPS operation was defined as the ability to activate and operate the digital pill, Reader, and smartphone app and successfully record digital pill ingestion. Finally, we discussed the potential technological challenges and user errors that participants may encounter and solutions to those barriers. The in-person portion of the training program lasted approximately 45 minutes.

Once the in-person portion of the DPS training program was completed, participants automatically entered the remote follow-up period of the program. In this portion, the study team programmed a series of 3 automated technology check-in text messages to be sent to each participant: the first at 24 hours following their in-person enrollment visit and then again on days 4 and 6. These messages asked participants to report via text message any technological barriers they were experiencing with the DPS (ie, by texting back 0 for “I need help with something” or 1 for “everything’s OK”). If technical assistance was requested in response to check-in messages, we attempted resolution via a standardized, hierarchical process, whereby resolution of all issues was first attempted via text message (through the web-based platform) and/or email with participants. If technical issues remained unresolved following attempts to rectify them via text message and/or email, a study team member escalated the issue to a phone call with participants for real-time evaluation and troubleshooting. Finally, participants were offered the opportunity to troubleshoot issues in person at the study site if they could not be resolved via any other means. Importantly, we did not offer advice through the training program regarding participants’ medication ingestion behaviors, device use, or feedback on their specific adherence (or nonadherence) trends. The DPS training program was considered complete at the end of the 6-day remote follow-up period. Study staff were available during the entire 90-day study period for additional technology-related concerns.

#### Qualitative User Experience Interviews

At the end of the 90-day study period, participants returned their DPS equipment and completed an individual, audio-recorded, semistructured qualitative interview. The aim of these interviews was to understand participants’ experiences operating the DPS and to obtain open-ended feedback on the DPS training program, including its perceived utility, adequacy for conveying the skills needed to operate the DPS, and suggested improvements to the training program. Interviewers were members of the study team who were trained in qualitative interviewing techniques (PRC, MJB, and YM).

### Data Analyses

#### Training Program Metrics

We assessed the successful completion of the DPS training program among participants (attending the initial in-person training session and responding to text messages during the remote follow-up portion), reasons for failure to complete the DPS training program, and the portion (in-person or remote) that was not completed. In addition, we recorded reasons participants did not complete the training program. We also logged all instances where participants contacted the study team during the training program period and over the course of the 90-day parent study and calculated basic descriptive statistics regarding engagement with the DPS training program, defined as the number of times individuals responded to a text message check-in.

#### Qualitative Analysis

All interviews were professionally transcribed and checked for errors. Three study team members trained in methods of qualitative analysis (GG, YM, and JN) read all transcripts individually to generate a qualitative codebook. The codebook was developed iteratively using the semistructured interview guide as a framework and consisted of both inductive and deductive themes. Team members reviewed the codebook throughout the development process, adding new codes and resolving discrepancies at each stage. Data related to participants’ feedback on the DPS training program were extracted and discussed as a group to develop important themes around user experience.

## Results

### Training Program Metrics

We enrolled 15 individuals during the study period (Table 1). Most participants used a smartphone with Apple iOS (n=9, 60%). The median age was 32 years (IQR 6.5; range 24-49). A total of 93% (n=14) of individuals reported being on PrEP before the start of the study, and 21% (3/14) of these participants reported missing at least two doses of PrEP over the previous 2 weeks. All individuals completed both the in-person and remote portions of the DPS training program.

**Table 1:** Demographics of study population (N=15).

Variables		Value
**Age (years)**		
	Median (IQR)	32 (6.5)
	Range	24-49
**Already prescribed PrEP^a^, n (%)**		
	Yes	14 (93)
	No	1 (7)
**Nonadherent to PrEP (via self-report)^b^, n (%)**		
	Yes	3 (21)
	No	11 (79)
**Own a smartphone, n (%)**		
	Android	6 (40)
	Apple	9 (60)

^a^PrEP: pre-exposure prophylaxis.

^b^Defined as missing ≥2 doses over the past 2 weeks. The denominator for this variable is 14, as 14 participants had already been prescribed pre-exposure prophylaxis and 1 had not.

**Figure 3:**
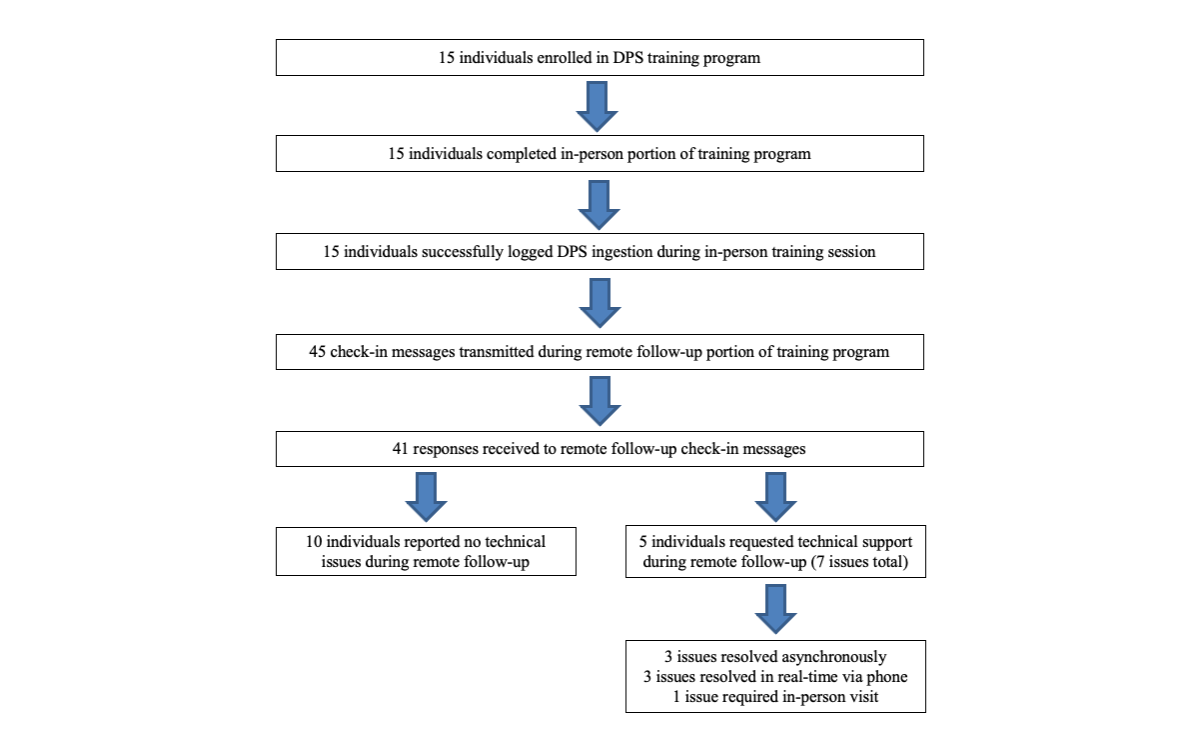
Participant enrollment and interaction with the digital pill system training program. DPS: digital pill system.

All participants enrolled in the training program were able to log an initial ingestion using the DPS during the in-person session and demonstrated the actual use of the DPS through recorded adherence events during the study period (Figure 3). During the remote text message follow-up portion of the training program, 67% (10/15) participants reported no issues with operating the DPS, whereas 33% (5/15) of participants requested technical assistance. Of those who requested technical assistance, 3 of the participants’ queries were resolved asynchronously via email or text message, 3 required real-time support through a telephone call, and 1 required in-person resolution. The nature of the technical challenges included difficulty connecting the Reader to the participant’s smartphone via Bluetooth, difficulty operating the mobile app, and technical questions related to charging and traveling with the Reader.

During the study period, we transmitted a total of 45 text messages to the 15 enrolled participants. Participants engaged with the DPS messages 91% (41/45 messages) of the time (ie, 41 messages were received from participants in response to DPS check-in messages). Of the 15 participants, 1 (7%) responded to just 1 of the 3 check-in messages, whereas 2 (13%) responded to 2 of the 3 messages, and 12 (80%) responded to all 3 messages. Of the 41 total responses received, 83% (n=34) of messages indicated that participants did not have difficulty operating the DPS, whereas 17% (n=7) of these messages included requests for technical assistance (a breakdown of message responses is given in Table 2).

**Table 2:** Characteristics of messaging and follow-up during the digital pill system training program.

Text message types			Value, n (%)
Total check-in messages transmitted during study period			45 (100)
**Responses received to check-in messages**			41 (91)
	Participants respondent to 1 of 3 check-in messages	1 (7)	
	Participants respondent to 2 of 3 check-in messages	2 (13)	
	Participants respondent to 3 of 3 check-in messages	12 (80)	
No difficulty operating the DPS^a^			34 (83)
**Request for technical assistance**			7 (17)
	Asynchronous resolution	3 (43)	
	Real-time telephone support from study team	3 (43)	
	In-person assessment of technical issue	1 (14)	

^a^DPS: digital pill system.

Of the messages requesting technical assistance, 43% (3/7) were successfully resolved asynchronously and 43% (3/7) were resolved in real time over the phone. One technical issue (1/7, 14%) required an in-person assessment, which was scheduled in addition to the existing study visits in the parent study. It was later determined that the participant had a malfunctioning mobile app and faulty Bluetooth permissions on their smartphone, which prevented successful pairing of the Reader with the phone. Once these issues were rectified, the participant was able to operate the DPS without any further problems. Although the study team remained available to troubleshoot further technological issues during the entire 90-day study period, no participants reported additional issues associated with the DPS after graduation from the training program.

### Qualitative User Experience Feedback

All participants completed a qualitative user experience interview at the end of the 90-day study period. Overall, the participants accepted the DPS training program. They reported that the information provided during the in-person portion of the program was clear, helpful, and adequate for understanding the purpose of the DPS and for troubleshooting minor technological issues. Participants perceived the remote text message follow-up portion of the training program to be highly valuable, as it reminded them to operate the DPS during their first week of use and enabled rapid communication with the study team in the event of technical issues. Participants also discussed a number of potential refinements that could be made to the DPS training program, including repeated refresher sessions on the DPS technology at each subsequent in-person visit (during the 90-day study period), as well as additional written materials to be used for self-guided troubleshooting of technological issues. Selected participant quotes from the qualitative user experience interviews are presented in Textbox 1.

**Qualitative user experience feedback (feedback content area and selected participant quotes).**

Experiences with the in-person portion of the digital pill system (DPS) training program

- “I thought that the first class and everything that happened that first day went really well, and you did a great job. [Study team member] made sure I understood everything… how to pair the phone, and how the technology worked, and how to keep track of it. There were clear instructions. There were lots of people standing by to answer questions if I ever had any questions after that” (age 29 years)
- “I think it was fast, efficient. It’s very easy. I think the hardest part would be just to get it set up, but you do that in the meeting, so that was easy” (age 24 years)
- “I think that was sufficient for me. I had no problem understanding the goals and how to interact with the device” (age 32 years)

Experiences with the remote portion of the DPS training program

- “I think I had one issue with repairing my device when my device and my phone disconnected, so I had to ask for the pairing code, but someone was available to respond over text for that. [Study team member] was always available over email, but I didn’t really even need to ask many follow up questions, because that first session was very thorough… I thought it was very, very helpful” (age 29 years)
- “The only thing was… I had the connectivity issues. I didn’t really know exactly what was going on. I think I had texted you, and you got back to me quickly, so I was able to figure it out” (age 40 years)

Suggested improvements to the DPS training program

- “I think it's helpful, but I think every visit, maybe you should go over it, or review it, or actually have the person bring in the technology and show you. That way, it's drilled into their head… I learn by doing, so the more I do something, the easier it becomes to learn… I think that would've been helpful to have written instructions or be in the email… I just forgot some of the things you told me” (age 32 years)
- “I think that someone else who might not be as technologically savvy might want more time, might want somebody to go through it more than once… but for me that was perfectly fine” (age 29 years)
- “I think the only information [to add] is… tools to make sure that the Reader will pick up [the ingestion] and things to look for if it’s not picking up, so the user or the person has ways to troubleshoot it or figure it out when it’s not working” (age 28 years)

## Discussion

### Principal Findings

The adoption of a DPS to measure medication adherence has important implications for clinical trials, pharmaceutical drug evaluation, and real-world challenges to adherence in various disease states. Although DPSs are designed to be relatively unobtrusive, the requirements for using novel capsule-based dosage forms containing ingestible sensors and a wearable Reader device that is paired with a smartphone may present challenges to adherence to the DPS. Similar to other novel technologies, training participants to correctly operate the DPS is an important first step toward obtaining reliable and accurate adherence data. Although previous investigations have described the feasibility of deploying a DPS among individuals to measure medication adherence, few studies have described best practices around training study participants to use the DPS [19,20]. This investigation is the first to describe a simple, scalable training program, grounded in the TAM, which teaches individuals how to operate the DPS and adhere to DPS instructions for use. MSM who participated in the study were able to accept and engage with the DPS training program. We anticipate that research groups who wish to adopt the DPS to measure adherence behavior in their participants can adapt this training program to standardize training procedures across enrolled participants or in the setting of clinical care.

For MSM, a DPS is acceptable as a method to measure PrEP adherence, but a critical gap remains in understanding how best to impart tools to help MSM operationalize DPS. MSM account for most of the new HIV infections in the United States, and co-occurring psychosocial epidemics of mental illness and substance use in MSM are associated with further increased HIV incidence [21,22]. People who use drugs are at an elevated risk for HIV because of high rates of both drug-related and sexual HIV risk behaviors. Neurocognitive impairment is also prevalent in this population and may serve as a barrier to optimal PrEP adherence. Together, these factors suggest that MSM who use substances face unique barriers to PrEP adherence and may benefit from technology-assisted adherence interventions such as the DPS [23-25]. Although other investigations have described the use of DPS in other disease states, this is the first study to provide a description of optimal training methods that imparts key skills in DPS operation in a standardized fashion. By standardizing training, this ensures that all critical factors associated with a successful DPS operation can be delivered to individuals using this technology.

Operationalizing the deployment of the DPS will ultimately depend on users’ experiences and acceptance of the tool, as well as robust methods for ensuring consistent use of the technology to receive actionable adherence data. By using the TAM to inform the development of our DPS training program, we were able to identify and understand participants’ perceived uses of the DPS, integrate potential barriers related to the intended uses of the technology into the program, and provide ongoing support for actual real-world use. The preliminary findings from our qualitative user experience interviews demonstrate that the training program was acceptable for teaching participants important skills associated with operating the DPS. Participants reported that both the in-person and remote follow-up components of the program were valuable for providing and reinforcing operational skills, as well as for efficient communication with the study team for the purpose of troubleshooting technological issues. Continued DPS refresher sessions, as well as enhanced written materials for resolving technological issues related to DPS use, were among participants’ proposed improvements to the training program. These data suggest that a variety of individuals can effectively and efficiently train future DPS users using this training program.

In the future, we anticipate that a training program such as that described here can be integrated into a starter package for DPS enrollment. Users may access the training program in a number of settings, including independently as part of a package sent directly to their homes or within a pharmacy while picking up their medications. Alternatively, in models where a DPS is used as a booster package when other adherence monitoring methods such as self-report or pharmacological measures have already detected nonadherence, this training program can capitalize on TAM pillars of perceived use and actual use to rapidly orient the user to the DPS and produce actionable data to improve adherence behavior. Finally, we intentionally designed the training program in such a way that it could be deployed by individuals with minimal training, to maximize its potential scalability. The study team members underwent a brief orientation to the DPS from the manufacturer (etectRx Inc) and received instructions on how to operate the system. After demonstrating competence in the DPS operation internally, they were cleared by the principal investigator to deliver the structured training program directly to the study participants.

The operational skills taught in this DPS training program can serve as a toolbox to help individuals who use these systems to measure adherence. Although we used this training program to teach operational skills to individuals using a DPS for the first time, periodic booster training programs may also be used to reinforce the use of the digital pill over the long term. The ideal structure of a DPS should include detailed plans for responding to nonadherence detected through the technology, whether in the form of in-person adherence assessments at clinical appointments, phone calls from care teams monitoring adherence behavior, or the automated messaging architecture to respond directly to DPS users following missed doses or changes in patterns of medication use. Ideally, any nonadherence-related assessments should also include an exploration of adherence to the DPS technology itself. Our data suggest that using a simple text message–based system can help detect potential technical difficulties associated with a DPS operation and can mitigate these barriers before participants’ disengagement with the technology. Even when significant technical issues arise that impair the ability of the DPS to measure and report adherence, a training program can quickly diagnose these issues and allow a research or clinical team to determine the level of support needed to intervene.

### Strengths and Limitations

This study had several limitations. First, we developed this DPS training program with a special focus on adherence to once-daily PrEP, and adherence to other medications may require specific and distinct instructions for integration with a DPS, to which PrEP-related guidelines may not be generalizable. However, we note that our training program can be easily modified to incorporate instructions specific to other xenobiotics. Second, we conducted the study at a single site with MSM who were young, well educated, and already engaged in health care services as well as research participation. The operation of a DPS may present specific barriers to other populations, especially those with less experience with novel technologies or who lack access to a smartphone. Third, the sample size of this preliminary pilot study was relatively small, and the findings may therefore not be representative of HIV-negative MSM who use substances. Finally, the parent study was conducted to understand the feasibility and acceptability of the DPS, and we therefore did not consider factors that would be required to integrate the training program of the DPS with clinical workflows.

### Conclusions

Overall, this study demonstrates that a brief DPS training program consisting of in-person instruction and asynchronous text message–based follow-up can identify potential technical issues associated with the initial use of a DPS to measure medication adherence. The training program is simple to implement, requires only a single in-person visit, and can be adapted as a tool to integrate the DPS into adherence strategies in various disease states. Future studies to evaluate the efficacy of this educational intervention in the deployment of this novel technology for specific patient populations are required.

## Abbreviations

DPS: digital pill system
MSM: men who have sex with men
NIH: National Institutes of Health
PrEP: pre-exposure prophylaxis
TAM: Technology Acceptance Model

## References

[1] Bandura A (2005). The primacy of self-regulation in health promotion. Appl Psychol.

[2] Haberer JE, Musinguzi N, Tsai AC, Boum 2nd Y, Bwana BM, Muzoora C, Hunt PW, Martin JN, Bangsberg DR (2017). Real-time electronic adherence monitoring plus follow-up improves adherence compared with standard electronic adherence monitoring. AIDS.

[3] Amico KR, Marcus JL, McMahan V, Liu A, Koester KA, Goicochea P, Anderson PL, Glidden D, Guanira J, Grant R (2014). Study product adherence measurement in the iPrEx placebo-controlled trial: concordance with drug detection. J Acquir Immune Defic Syndr.

[4] Anderson P, Glidden DV, Liu A, Buchbinder S, Lama JR, Guanira JV, McMahan V, Bushman LR, Casapía M, Montoya-Herrera O, Veloso VG, Mayer KH, Chariyalertsak S, Schechter M, Bekker LG, Kallás EG, Grant RM, iPrEx Study Team (2012). Emtricitabine-tenofovir concentrations and pre-exposure prophylaxis efficacy in men who have sex with men. Sci Transl Med.

[5] Closson E, Mitty JA, Malone J, Mayer KH, Mimiaga MJ (2018). Exploring strategies for PrEP adherence and dosing preferences in the context of sexualized recreational drug use among MSM: a qualitative study. AIDS Care.

[6] Anand T, Nitpolprasert C, Ananworanich J, Pakam C, Nonenoy S, Jantarapakde J, Sohn AH, Phanuphak P, Phanuphak N (2015). Innovative strategies using communications technologies to engage gay men and other men who have sex with men into early HIV testing and treatment in Thailand. J Virus Erad.

[7] Turner D, Lockhart E, Marhefka SL (2019). Willingness of MSM living with HIV to take part in video-groups: application of the Technology Readiness and Acceptance Model. AIDS Behav.

[8] Saberi P, Rose CD, Wootton AR, Ming K, Legnitto D, Jeske M, Pollack LM, Johnson MO, Gruber VA, Neilands TB (2020). Use of technology for delivery of mental health and substance use services to youth living with HIV: a mixed-methods perspective. AIDS Care.

[9] Steinkamp JM, Goldblatt N, Borodovsky JT, LaVertu A, Kronish IM, Marsch LA, Schuman-Olivier Z (2019). Technological interventions for medication adherence in adult mental health and substance use disorders: a systematic review. JMIR Ment Health.

[10] Browne SH, Behzadi Y, Littlewort G (2015). Let visuals tell the story: medication adherence in patients with Type II diabetes captured by a novel ingestion sensor platform. JMIR Mhealth Uhealth.

[11] Carreiro S, Smelson D, Ranney M, Horvath KJ, Picard RW, Boudreaux ED, Hayes R, Boyer EW (2015). Real-time mobile detection of drug use with wearable biosensors: a pilot study. J Med Toxicol.

[12] Chai PR, Carreiro S, Innes BJ, Chapman B, Schreiber KL, Edwards RR, Carrico AW, Boyer EW (2017). Oxycodone ingestion patterns in acute fracture pain with digital pills. Anesth Analg.

[13] Chai P, Carreiro S, Innes BJ, Rosen RK, O'Cleirigh C, Mayer KH, Boyer EW (2017). Digital pills to measure opioid ingestion patterns in emergency department patients with acute fracture pain: a pilot study. J Med Internet Res.

[14] Chai PR, Castillo-Mancilla J, Buffkin E, Darling C, Rosen RK, Horvath KJ, Boudreaux ED, Robbins GK, Hibberd PL, Boyer EW (2015). Utilizing an ingestible biosensor to assess real-time medication adherence. J Med Toxicol.

[15] Davis FD (1989). Perceived usefulness, perceived ease of use, and user acceptance of information technology. MIS Q.

[16] Venkatesh V, Morris MG, Davis GB, Davis FD (2003). User acceptance of information technology: toward a unified view. MIS Q.

[17] Venkatesh V, Bala H (2008). Technology acceptance model 3 and a research agenda on interventions. Decis Sci.

[18] Chai P, Goodman G, Bustamante M, Mendez L, Mohamed Y, Mayer KH, Boyer EW, Rosen RK, O'Cleirigh C (2020). Design and delivery of real-time adherence data to men who have sex with men using antiretroviral pre-exposure prophylaxis via an ingestible electronic sensor. AIDS Behav.

[19] Daar ES, Rosen MI, Wang Y, Siqueiros L, Shen J, Guerrero M, Xiong D, Dao J, Young T, Corado K, Fletcher CV, Liu H (2020). Real-time and wireless assessment of adherence to antiretroviral therapy with co-encapsulated ingestion sensor in hiv-infected patients: a pilot study. Clin Transl Sci.

[20] Peters-Strickland T, Hatch A, Adenwala A, Atkinson K, Bartfeld B (2018). Human factors evaluation of a novel digital medicine system in psychiatry. Neuropsychiatric Disease Treat.

[21] (2018). HIV surveillance reports. Centers for Disease Control and Prevention.

[22] Mimiaga MJ, OʼCleirigh C, Biello KB, Robertson AM, Safren SA, Coates TJ, Koblin BA, Chesney MA, Donnell DJ, Stall RD, Mayer KH (2015). The effect of psychosocial syndemic production on 4-year HIV incidence and risk behavior in a large cohort of sexually active men who have sex with men. J Acquir Immune Defic Syndr.

[23] Hoenigl M, Jain S, Moore D, Collins D, Sun X, Anderson PL, Corado K, Blumenthal JS, Daar ES, Milam J, Dubé MP, Morris S, California Collaborative Treatment Group 595 Team (2018). Substance use and adherence to HIV preexposure prophylaxis for men who have sex with men. Emerg Infect Dis.

[24] Sanborn V, Gunstad J, Shrestha R, Mistler CB, Copenhaver MM (2020). Cognitive profiles in persons with opioid use disorder enrolled in methadone treatment. Appl Neuropsychol Adult.

[25] Shrestha R, Karki P, Altice FL, Huedo-Medina TB, Meyer JP, Madden L, Copenhaver M (2017). Correlates of willingness to initiate pre-exposure prophylaxis and anticipation of practicing safer drug- and sex-related behaviors among high-risk drug users on methadone treatment. Drug Alcohol Depend.

